# YhcB mediates growth-phase control of fatty acid biosynthesis through regulation of acetyl-CoA carboxylase

**DOI:** 10.1128/mbio.03681-25

**Published:** 2026-01-26

**Authors:** Hannah M. Stanley, M. Stephen Trent

**Affiliations:** 1 Department of Microbiology, College of Art and Sciences, University of Georgia 189270, Athens, Georgia, USA; 2 Department of Infectious Diseases, College of Veterinary Medicine, University of Georgia 70734, Athens, Georgia, USA; NYU Langone Health, New York, New York, USA

**Keywords:** YhcB, ACC complex, acetyl-CoA carboxylase, Fatty acid biosynthesis, cell envelope, membrane biogenesis, AccA

## Abstract

Cell envelope biogenesis is an essential process that requires coordination of many complex pathways, including the synthesis of fatty acids, lipopolysaccharides, and glycerophospholipids. Loss of *Escherichia coli* YhcB has been demonstrated to result in filamentous cell morphology and membrane defects due to overactive fatty acid biosynthesis, a phenotype that intensifies in early stationary phase when lipid biosynthesis is normally downregulated. In bacteria, fatty acid biosynthesis is initiated by the acetyl-coenzyme A (CoA) carboxylase (ACC) complex, which catalyzes the carboxylation of acetyl-CoA to malonyl-CoA, the first and key regulatory step in the pathway. Here, we show that YhcB interacts with AccA, one of four subunits of the ACC complex. This interaction is growth-phase dependent, occurring specifically during the transition from exponential to stationary phase. We also report that AccA undergoes proteolytic degradation, a process that is modulated by YhcB, but not strictly dependent upon its presence. While YhcB is not required for AccA degradation, YhcB and AccA interactions correlate with reduced fatty acid biosynthesis in the early stationary phase. These findings indicate that YhcB may function by sequestering AccA, thereby inhibiting ACC activity. Together, our findings suggest two previously unrecognized mechanisms for regulating ACC activity: targeted proteolysis of AccA and its sequestration by YhcB.

**IMPORTANCE:**

The gram-negative cell envelope is synthesized through coordination of many complex pathways, requiring adaptable regulation at the DNA, RNA, and protein levels. Fatty acid biosynthesis, a highly energy-demanding process, is essential for cell viability and a promising target for antimicrobial design. We identify two previously unrecognized mechanisms that regulate this pathway: proteolytic turnover and YhcB-mediated sequestration of a protein dedicated to the initiation of fatty acid biosynthesis. These findings reveal new layers of control over membrane biogenesis that could be exploited for antimicrobial strategies.

## INTRODUCTION

The outer membrane (OM) of the gram-negative bacterial cell envelope is a selectively permeable, asymmetric barrier that allows the cell to withstand a variety of environments and chemical stresses. This unique structure is composed of glycerophospholipids (GPLs) in the inner leaflet and primarily lipopolysaccharide (LPS) in the outer leaflet. A peptidoglycan cell wall is tightly associated with the OM in the periplasm, a compartment that separates the OM and the cytoplasmic inner membrane (IM), which is comprised of GPLs (1). Construction and maintenance of this highly organized envelope requires precise control over many pathways, including the biosynthesis and distribution of its lipid components.

At the core of membrane biogenesis is the synthesis of fatty acids that serve as essential building blocks of both GPLs and LPS. *Escherichia coli* produces fatty acids through the type II fatty acid biosynthesis pathway (FAB), a modular system of discrete enzymes. This pathway is initiated by the acetyl-coenzyme A (CoA) carboxylase (ACC) complex through the generation of malonyl-CoA, a product dedicated solely to FAB. The ACC complex is composed of four unique subunits, AccABCD, that catalyze two half-reactions. AccC (biotin carboxylase) carboxylates the biotin attached to the biotin carrier protein (AccB), and the carboxyltransferase (α subunit AccA and β subunit AccD) transfers this carboxyl group from biotin to acetyl-CoA (2).

The resulting malonyl-CoA is then transferred onto acyl carrier protein (ACP), a small, highly conserved cofactor that tethers the growing fatty acyl chain. During chain elongation, the fatty acid is lengthened by two carbons each time. This iterative process requires a series of condensation, dehydration, and reduction phases that are carried out by numerous FAB enzymes. Most fatty acids (i.e., acyl-ACPs) synthesized through FAB are used downstream to generate GPLs and LPS, with the majority being incorporated into GPLs. Notably, GPL synthesis can also utilize exogenous fatty acids that have been converted into acyl-CoAs of the appropriate chain length but not yet consumed by the fatty acid degradation (FAD) pathway. However, with few exceptions, LPS biosynthesis is specific for acyl-ACP donors and does not use exogenous fatty acids (3, 4). Thus, the pathways for GPL and LPS assembly require not only a common pool of fatty acids but also careful regulation of their chemical form and abundance.

Although the enzymatic steps of fatty acid biosynthesis have been well defined for decades, how cells regulate flux through this essential and energy-intensive pathway remains unclear. Known mechanisms include transcriptional control by the global regulators FadR and FabR, as well as feedback inhibition of ACC by accumulated long-chain acyl-ACP species (5). It has also been reported that the stringent response alarmone ppGpp inhibits membrane lipid synthesis, primarily through effects on downstream enzymes such as PlsB that are required for GPL biogenesis (6, 7). However, none of these mechanisms fully explains how cells dynamically adjust fatty acid synthesis to accommodate the dramatic changes in membrane demands during different growth phases and stress conditions.

Recent progress toward understanding how LPS biosynthesis is regulated has come from studies of the essential protease FtsH that, in conjunction with YejM and LapB, controls cellular levels of LpxC—the enzyme that catalyzes the committed step of LPS synthesis (8–11). The existence of such a complex, multi-protein regulatory mechanism suggests that other membrane biogenesis pathways may also be controlled similarly. Therefore, the persistent gap in our understanding of FAB regulation remains a central, unresolved question in bacterial physiology.

In a prior study, we examined the role of YhcB based on the effects caused by its loss. Preliminary characterization of the *yhcB* mutant in *E. coli* K-12 strain W3110 demonstrated increased sensitivity to antibiotics, filamentous cell morphology, and accumulation of cell debris; these findings were consistent with prior reports (12–15). To explore these defects further, we conducted suppressor screens and short-term evolution experiments. We found that mutations in genes encoding subunits of the essential ACC complex dominated the *yhcB* evolved populations and fully suppressed *yhcB* defects. Similarly, mutations in *acc* genes restored the viability of a synthetically lethal *lpxM*, *yhcB* double mutant. LpxM acts as a late acyltransferase in the synthesis of the lipid A anchor of LPS.

With this mechanistic insight, we turned our attention to the role of YhcB in membrane biogenesis. By monitoring changes in lipid synthesis, we determined that the loss of YhcB results in increased GPL production and alteration of the GPL-to-LPS ratio. Transferring the *accA*_R175L_ allele isolated from the suppressor screen into the parent *yhcB* mutant fully rescued the cell morphology and GPL-to-LPS ratio defects. We found that this genetic rescue of the *yhcB* mutant could be recapitulated by growing *yhcB* in the presence of cerulenin, a FAB inhibitor (12).

These data demonstrated that FAB flux (and therefore GPL flux) in the *yhcB* mutant is aberrantly increased. Only reducing FAB flux could fully rescue the *yhcB* mutant (12). With a better understanding of the defects that result in the absence of YhcB, we therefore focused our investigation on the role YhcB plays in the large, complex system of membrane biogenesis. Here, we find that YhcB has an early stationary phase role, revealing why defects in the mutant are exacerbated at this phase of growth. We report that YhcB and AccA of the ACC complex directly interact when probed from both directions and that this interaction is detected only in the early stationary phase. Notably, we found that AccA is proteolytically degraded in the exponential phase and that protein abundance significantly decreases from the exponential to the early stationary phase. While AccA and YhcB levels are inversely correlated, the reduction of AccA levels is not dependent on YhcB. However, modulating YhcB levels in the cell affects AccA abundance. We therefore conclude that two novel methods of regulation of ACC occur during the transition to the stationary phase, both of which are influenced by the presence of YhcB.

## RESULTS

### YhcB is required during the early stationary phase

In a prior study, we found that the loss of *yhcB* results in filamentation, cell debris, and impaired growth that is particularly evident in the early stationary phase (12). To define the temporal role of YhcB during growth, we used CRISPR interference (CRISPRi) to deplete *yhcB* transcripts at key intervals of growth. CRISPRi employs a catalytically dead Cas9 (dCas9) and a customized guide RNA to block transcription in a targeted manner (16). For these experiments, we constructed a strain with chromosomal *dcas9* under control of a pTet promoter, which is inducible by anhydrous tetracycline (aTc), and a plasmid carrying the small guide RNA (sgRNA) under the control of an isopropyl β-D-1-thiogalactopyranoside (IPTG)-inducible promoter. This design allowed us to assess the growth phases in which loss of YhcB triggers filamentation.

**Fig 1:**
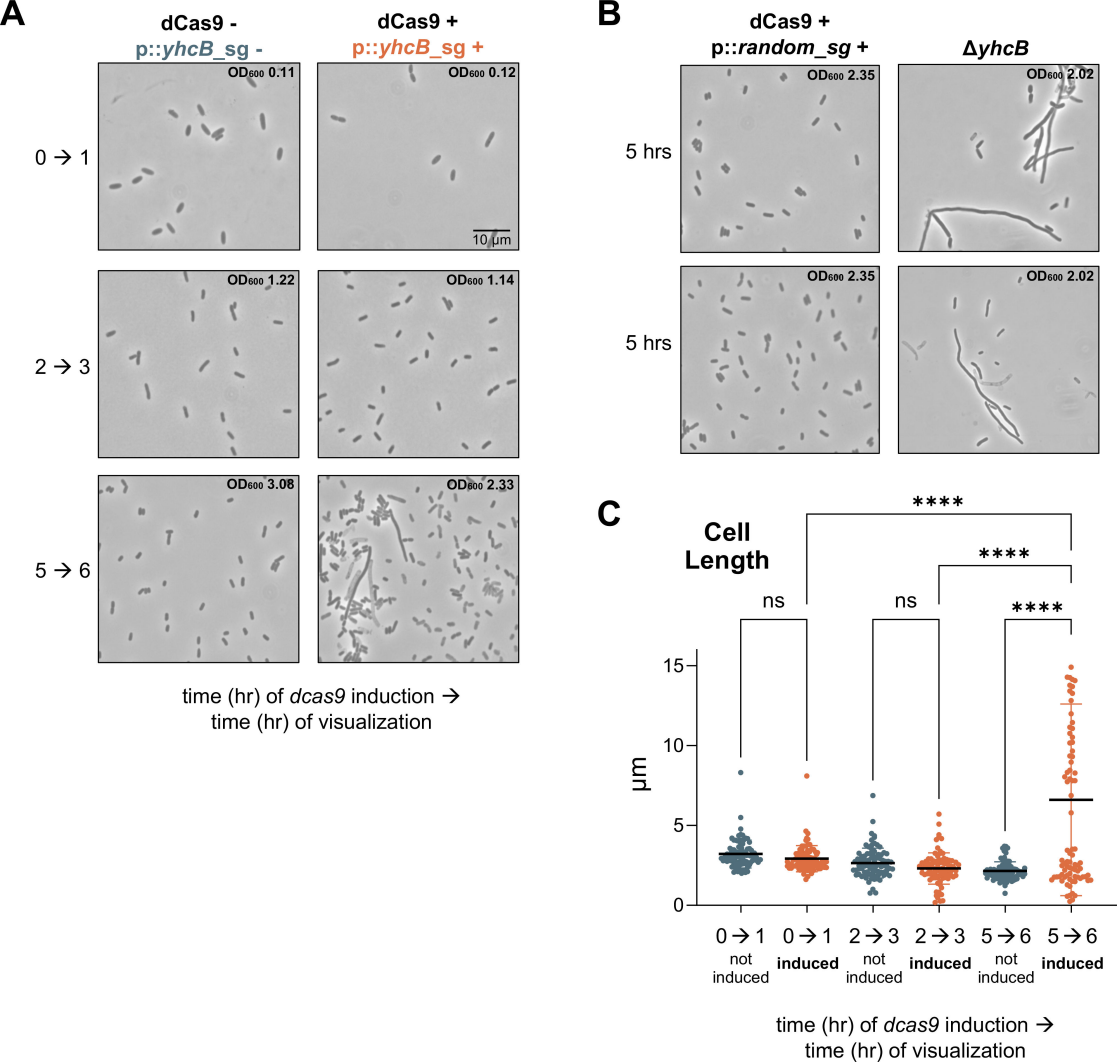
YhcB is required during the transition into the stationary phase. (**A**) Microscopy of cells during knockdown of *yhcB* in different phases of growth. Phase-contrast microscopy at 1,000× magnification of the indicated cells with a 10-µm scale bar is shown. Strains harbor a chromosomal copy of *dcas9* under the control of an aTc-inducible promoter and a vector carrying a small guide targeting *yhcB* under the control of an IPTG-inducible promoter. Strains were back-diluted to OD_600_ 0.05 either with 1 mM IPTG (*yhcB* sg +) or without (*yhcB* sg −). Expression of *dcas9* was induced with 200 nM aTc at the indicated time. The numbers on the left side of the arrow indicate the time (h) when the expression of the *dcas9* was induced (dCas9 +/−), and the numbers to the right of the arrow indicate when those strains were visualized (h). Representative OD_600_ measurements at the time of microscopy for each strain are indicated in the top right of the micrographs. See Fig. S1 for related data. (**B**) Microscopy at 5 h post-back-dilution of the induced, randomized small guide control strain (dCas9 + and *random*_sq +) and the *yhcB* mutant (no aTc or IPTG added) is included for comparison. Two micrographs are shown for each strain, and representative OD_600_ measurements at the time of microscopy are indicated. (**C**) Cell size analysis was performed on 87 cells per strain with MicrobeJ, and measurements were assessed through a one-way analysis of variance (ANOVA) test with Brown-Forsythe and Welch tests assuming that standard deviations were not equal. Significant differences were assessed using a Games-Howell test. NS indicates not significant; *P ≤ 0.05; **P ≤ 0.01; ***P ≤ 0.001; ****P ≤ 0.0001. Data shown are representative of two biological replicates.

The *yhcB* sgRNA was induced in simultaneously started cultures, and dCas9 was induced sequentially every hour for 5 h in independent cultures (Fig. 1A; see Fig. S1A for additional time points and controls). A noninduced control was included at each time point, and dCas9 expression alone did not cause cells to filament (Fig. 1B). Cells were visualized using phase-contrast microscopy to assess cell size. In the exponential phase (0-to-1 and 2-to-3 time points), inhibition of *yhcB* expression for 1 h did not cause filamentation as seen in Δ*yhcB* (Fig. 1B). When *yhcB* transcription was inhibited at the 5-to-6-h time point, the cells filamented within 1 h of loss of *yhcB* transcripts. Uninduced controls did not filament at any timepoint.

Cell size analysis indicated that the *yhcB* knockdown did not cause significant filamentation until the 5-to-6-h timepoint (Fig. 1C; see Fig. S1B for additional timepoints). These results suggest that the phenotypes seen in the *yhcB* mutant at the stationary phase are not due to a cumulative effect of YhcB loss but instead are due to a time-phase-specific effect. Thus, YhcB appears to be dispensable for maintaining cell shape in the exponential phase but is critical in the early stationary phase when membrane biogenesis must adjust to reduced growth.

### Loss of YhcB does not cause transcriptional changes in genes involved in fatty acid or GPL synthesis

**Fig 2:**
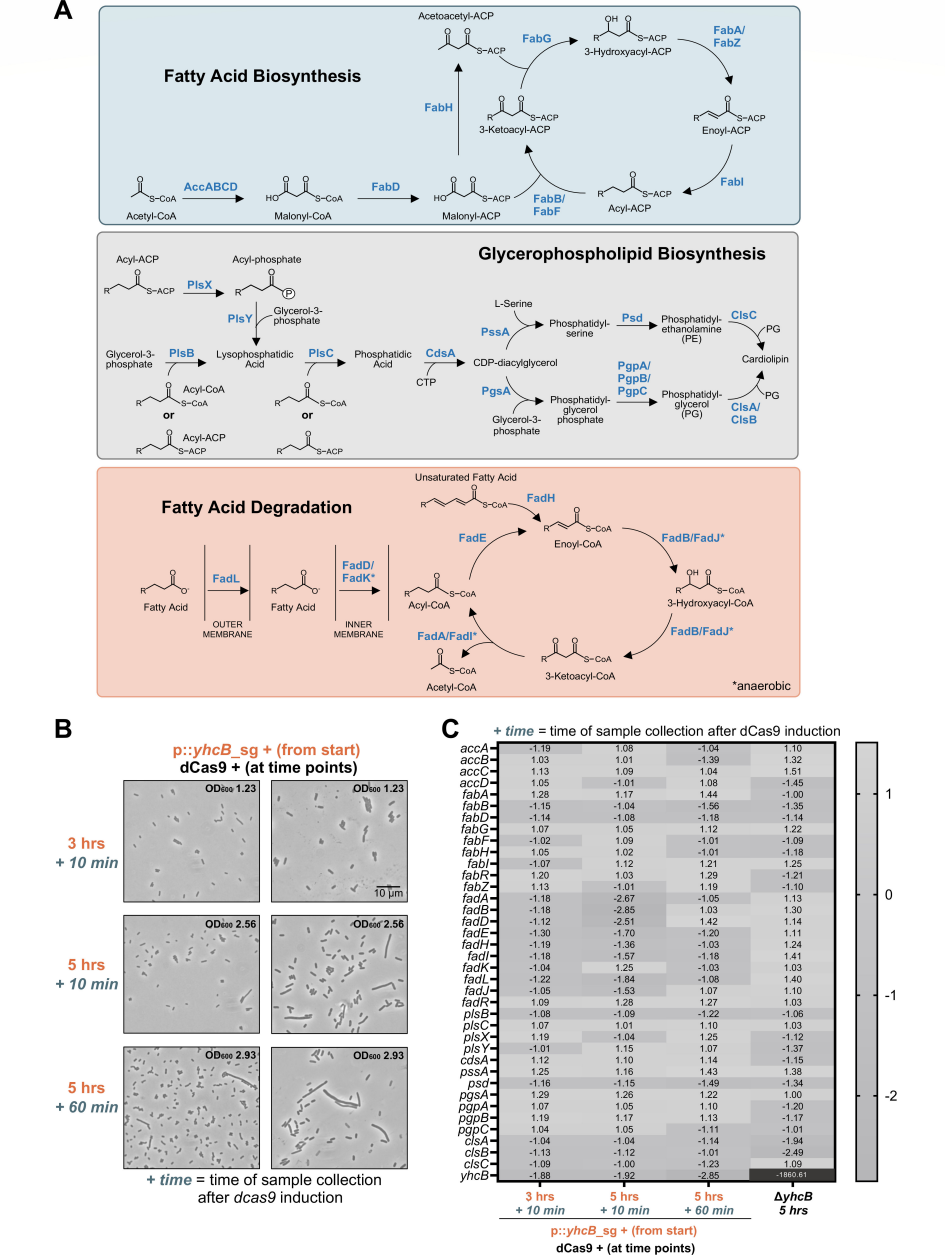
Stationary-phase defects in Δ*yhcB* occur without transcriptional changes. (**A**) Pathways of fatty acid biosynthesis, GPL biosynthesis, and FAD are shown. The * indicates anaerobic β-oxidation enzymes in the FAD pathway. (**B**) Phase-contrast microscopy at 1,000× magnification with a 10-µm scale bar of a strain with a chromosomal copy of *dcas9* under the control of an aTc-inducible promoter and a vector carrying a small guide targeting *yhcB* under the control of an IPTG-inducible promoter. Strains were back-diluted to OD_600_ 0.05 either with 1 mM IPTG (*yhcB* sg +) or without (*yhcB* sg −; see Fig. S2). Expression of *dcas9* was induced with 200 nM aTc at the indicated times. Numbers on the left side of the micrographs indicate the time when expression of *dcas9* was induced, and the italicized numbers indicate when cells were visualized and harvested for RNA-Seq. Representative OD_600_ measurements at the time of microscopy for the induced sg + strain are indicated in the top right of the micrographs. Two micrographs are shown per sample. See Fig. S2 for related data. (**C**) RNA-Seq of induced CRISPRi strains with the *yhcB-*targeted small guide was compared to a randomized small guide control. A *yhcB* mutant harvested at 5 h (OD_600_ ~2.0) was compared to the WT harvested at the same time. Weighted fold changes of transcripts of genes involved in fatty acid biosynthesis and degradation, and GPL biosynthesis are displayed. The numbers on the *x*-axis correspond to the timepoints shown in panel A. Data include biological triplicates.

We hypothesized that the filamentation and lipid overproduction phenotypes of the *yhcB* mutant might arise from transcriptional dysregulation of FAB, FAD, and GPL synthesis pathways (Fig. 2A). To test this hypothesis, we combined the CRISPRi approach with global transcript sequencing, RNA-Seq. Two sgRNAs were used: one targeting the promoter region of *yhcB* and the other, a randomized guide demonstrated by Cui and co-authors (16) to act as a control for off-target effects. Strains were grown with induced small guide expression, and dCas9 was induced at 3 or 5 h. Cells were grown an additional 10 or 60 min (only for the 5-h timepoint) prior to RNA-Seq sample collection. Microscopy at these timepoints demonstrates that cells begin to filament within 10 min of *yhcB* expression knockdown after 5 h of growth and worsen with 60 min of knockdown. Cells at the 3-h timepoint are not filamentous (Fig. 2B; see Fig. S2 for additional controls).

Transcripts of the induced, *yhcB-*small-guide samples were compared against the induced, randomized-small-guide control samples at each of the three timepoints. RNA-Seq of the *yhcB* mutant compared to the WT at 5 h was also performed (Fig. 2C). Comparison of these data sets (Data Set S1) revealed no significant changes in the expression of genes encoding FAB, FAD, or GPL enzymes (Fig. 2A) that could explain the cell filamentation or overactive FAB phenotypes in the *yhcB* knockdown or *yhcB* mutant strains (12). These data suggest that YhcB affects fatty acid flux through a post-transcriptional mechanism.

### YhcB directly interacts with AccA

**Fig 3:**
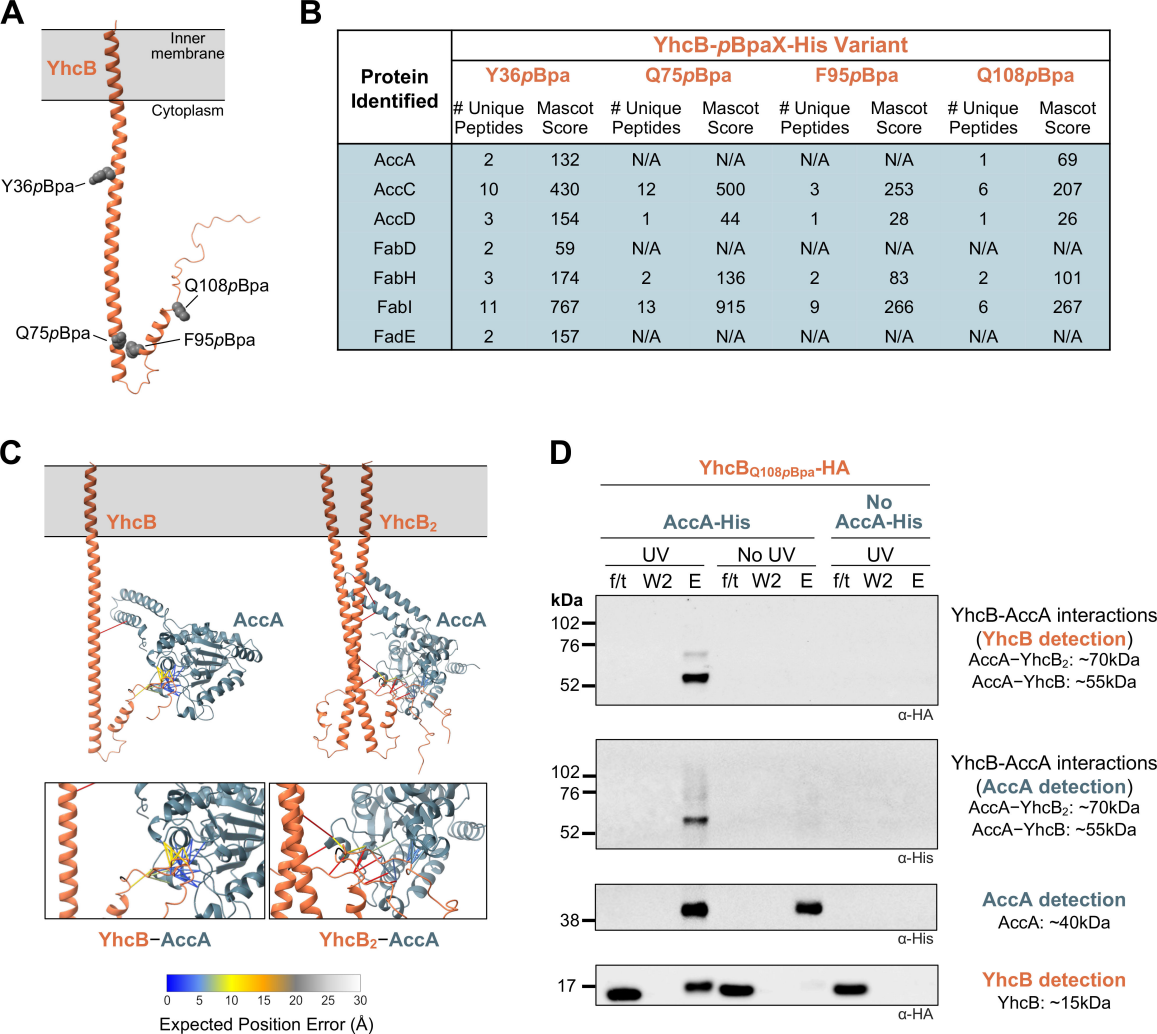
YhcB and AccA interact. (**A**) AlphaFold 3 model of the YhcB monomer. The transmembrane domain of YhcB is colored a dark orange, and the residues (Y36, Q75, F95, and Q108) mutated to the photoactivatable crosslinker *p*-benzoyl-L-phenylalanine (*p*Bpa) are shown as space-fill in gray. (**B**) Mass spectrometry analysis of elutions from ultraviolet (UV)-crosslinked, His-purified *p*Bpa-YhcB variants. Expression of each *p*Bpa-*yhcB* mutant and the *p*Bpa-tRNA synthetase was controlled via arabinose-inducible promoters on two separate plasmids. Eluted proteins were precipitated with trichloroacetic acid (TCA), subjected to proteolytic digestion, and the resulting peptides identified via liquid chromatography tandem mass spectrometry (LC-MS/MS). Proteins not only present in the crosslinked samples but also present in either the non-crosslinked or empty vector controls were excluded. For each protein, the number of unique peptides and the Mascot score are provided. (**C**) AlphaFold 3 models of AccA and YhcB (1:1 and 1:2 stoichiometries, as seen in panel D) with predicted interactions ≤5 Å or less. Scale bar represents expected position error (in Å) of predicted interactions, where bright blue indicates a strong confidence and white predicts a weak confidence. (**D**) Western blot images of His pulldowns using chromosomally tagged AccA-His as bait and chromosomally tagged YhcB-HA as prey. A non-His-tagged control and a no UV control are included. Blots were probed using anti-HA and anti-His antibodies. Representative OD_600_ measurements at the 5-h timepoint were as follows: 2.46 for AccA-His YhcB_Q108_*_p_*_Bpa_ and 2.53 for YhcB_Q108_*_p_*_Bpa_. Data are representative of a minimum of three biological replicates.

Because there were no large-scale transcriptional changes that could have caused the overactive FAB phenotypes present in the *yhcB* mutant, we next investigated the protein interactions of YhcB. To ensure capture of potentially transient interactions, the photoreactive amino acid *p*-benzoyl-L-phenylalanine (*p*Bpa) was used. This photocrosslinking amino acid has been demonstrated to be an efficient crosslinker capable of capturing interactions that occur within close proximity to the photoactivatable residue (17, 18). Fourteen large or aromatic residues in YhcB were chosen for conversion to *p*Bpa via site-directed mutagenesis and encoded on a plasmid under the control of an arabinose-inducible promoter. Utilizing the vector-based *p*Bpa aminoacyl-tRNA synthetase/tRNA pair system developed by Chin and co-authors (19), the individual C-terminally His-tagged *p*Bpa variants were expressed in the presence of exogenously provided *p*Bpa. Each variant was tested for its ability to complement the *yhcB* mutant (Fig. S3). Four *p*Bpa variants (residues Y36, Q75, F95, and Q108) were then chosen for use in future experiments based on their distribution throughout the protein and ability to complement the mutant (Fig. 3A).

Because YhcB has an early stationary phase role, cells were grown for 5 h prior to exposure to UV light. The samples were lysed, solubilized in detergent, and passed over a gravity column containing nickel-nitriloacetic acid (Ni-NTA) resin (see Materials and Methods for details). The elution fractions were precipitated using TCA prior to peptide identification via LC-MS/MS. An empty vector control (no YhcB-His) and a non-crosslinked control were included to filter out non-specific proteins that bound to the column. Proteins appearing in all elutions were removed from analysis. The remaining proteins were analyzed for connections to the overactive FAB phenotypes displayed in the *yhcB* mutant (Fig. 3B).

Proteins related to FAB and FAD that were pulled down with YhcB included AccA, AccC, AccD, FabD, FabH, FabI, and FadE. AccA, AccC, and AccD were of particular interest because mutations in the genes encoding these proteins were isolated from suppressors that rescued viability of the *yhcB lpxM* double mutant and stabilized populations of the *yhcB* mutant in a long-term evolution experiment (12). A suppressor mutation in *accA* (R175L) was capable of fully suppressing a *yhcB* mutant, rescuing the filamentation defect by slowing FAB (12). Given the strong phenotype associated with the ACC complex in the *yhcB* mutant, we further assessed potential protein interactions between YhcB and AccA, AccB, AccC, and AccD using the artificial intelligence protein structure model AlphaFold 3 (Fig. 3C; Fig. S4) (20, 21). Proposed interactions within 5 Å were analyzed using ChimeraX (22). Interactions between YhcB and AccB, AccC, and AccD were less confident than those interactions predicted between YhcB and AccA (Fig. S4; Fig. 3C). Because it was likely that some ACC subunits pulled down due to interactions within the complex and not due to specific interaction with YhcB, we focused on AccA due to the higher confidence in the predicted interactions between the two proteins.

We validated the interactions between YhcB and AccA through a pulldown experiment where AccA was the bait, and YhcB was the prey. In this case, we used a strain chromosomally expressing wild-type AccA containing a His-tag and the YhcB_Q108_*_p_*_Bpa_ variant containing an HA-tag. Given that YhcB is an IM protein, the membrane fraction was used as the input for this experiment to enrich for any AccA-YhcB complexes. The flow-through, final wash, and elution fractions were resolved using SDS-PAGE and probed via Western blot. A non-crosslinked control and a strain without a His-tagged AccA were included as controls. Two bands at approximately 55 and 70 kilodaltons (kDa) are detectable only in the UV-exposed samples when AccA-His is present (Fig. 3D). These crosslinked bands correspond to complexes of 1AccA:1YhcB and 1AccA:2YhcB. Altogether, the reciprocal pulldown experiments (Fig. 3B and D) and the supporting AlphaFold predictions (Fig. 3C) clearly show that YhcB and AccA have strong interactions within the cell.

### YhcB stationary phenotypes correlate with AccA interactions

**Fig 4:**
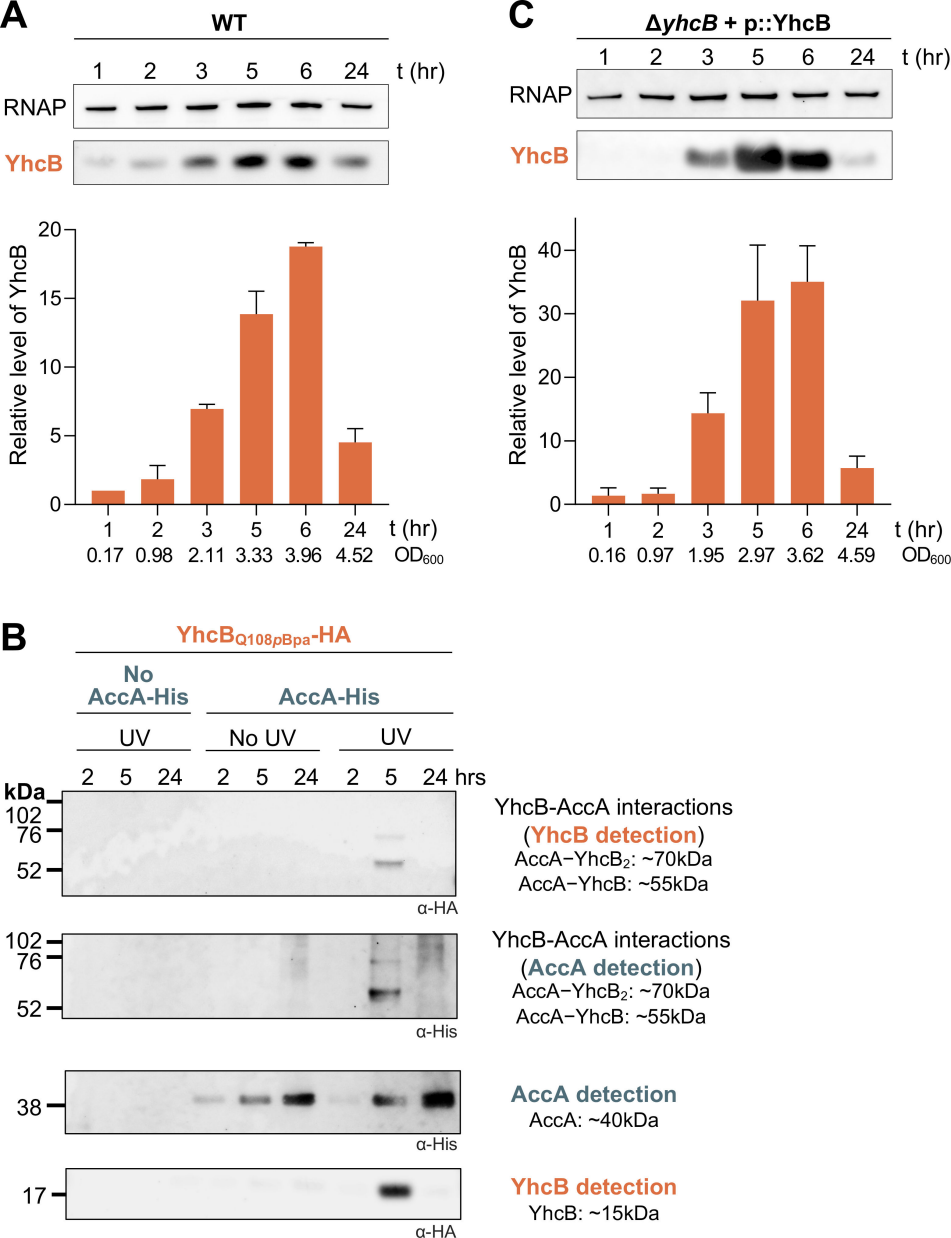
YhcB stationary-phase phenotypes correlate with AccA interactions. (**A**) Steady-state assay of YhcB abundance 1, 2, 3, 5, 6, and 24 h post-inoculation. Samples were normalized by total protein, and 1.5 µg was loaded per lane. YhcB was detected by Western blot using anti-His antibody, and RNAP was detected by anti-RNAP antibody and served as a loading control. YhcB abundance was quantified by densitometry, with the chromosomal YhcB sample at the 1-h mark set to 1.0 and all values normalized to RNAP. Representative OD_600_ measurements of WT (YhcB-HA) for each timepoint are provided. (**B**) His pulldowns used chromosomally tagged AccA-His as bait and chromosomally tagged YhcB-HA as prey. Samples were UV-crosslinked at 2, 5, and 24 h and probed with anti-HA and anti-His antibodies. Representative OD_600_ measurements of AccA-His YhcB_Q108_*_p_*_Bpa_ were 0.51 (2 h), 2.46 (5 h), and 3.72 (24 h). Representative OD_600_ measurements of YhcB_Q108_*_p_*_Bpa_ were 0.54 (2 h), 2.53 (5 h), and 3.51 (24 h). (**C**) Steady-state assay of YhcB abundance during ectopic expression from an arabinose-inducible promoter (0.05% arabinose). YhcB abundance was assessed as described in panel A. Representative OD_600_ measurements of Δ*yhcB* + p::YhcB for each timepoint are provided. Data shown are representative of a minimum of three biological replicates.

To better understand the stationary phase role of YhcB, steady-state assays of a chromosomally encoded YhcB-HA were performed. Samples were taken at 1, 2, 3, 5, 6, and 24 h and normalized by protein content prior to resolution via SDS-PAGE and visualization via Western blot. Relative YhcB abundance was normalized to a loading control, RNA polymerase β (RNAP). YhcB levels are lowest in the exponential phase and highest in the early stationary phase (Fig. 4A). By the late stationary phase, YhcB levels have decreased to an intermediate level similar to what is seen at the 2- and 3-h timepoints.

We wondered whether the interactions between YhcB and AccA were part of the early stationary phase role of YhcB. Therefore, we again probed for YhcB_Q108_*_p_*_Bpa_/AccA interactions in UV-crosslinked samples after 2, 5, and 24 h of growth (Fig. 4B). The interactions between YhcB and AccA were only detectable during early stationary phase at the peak of YhcB levels. However, the question of whether YhcB and AccA were still interacting at the other two timepoints but simply undetectable remained. To determine whether overexpression of YhcB could be used to probe for YhcB-AccA interactions at timepoints beyond those dictated by native regulation, we assessed steady-state levels of YhcB in a complemented *yhcB* mutant (Fig. 4C). Expression of YhcB from this vector was controlled by an arabinose-inducible promoter, not the native *yhcB* promoter, yet the trend of YhcB abundance was identical to the one seen in the native-controlled YhcB steady state assay (Fig. 4A). YhcB has been suggested to be growth-phase regulated by the small RNA SdsR, which is present in the stationary phase (23, 24). The mediator of exponential-phase control of YhcB remains unclear. However, YhcB levels—and therefore YhcB-AccA interactions—are growth-phase-dependent.

### AccA is growth phase regulated through proteolysis

**Fig 5:**
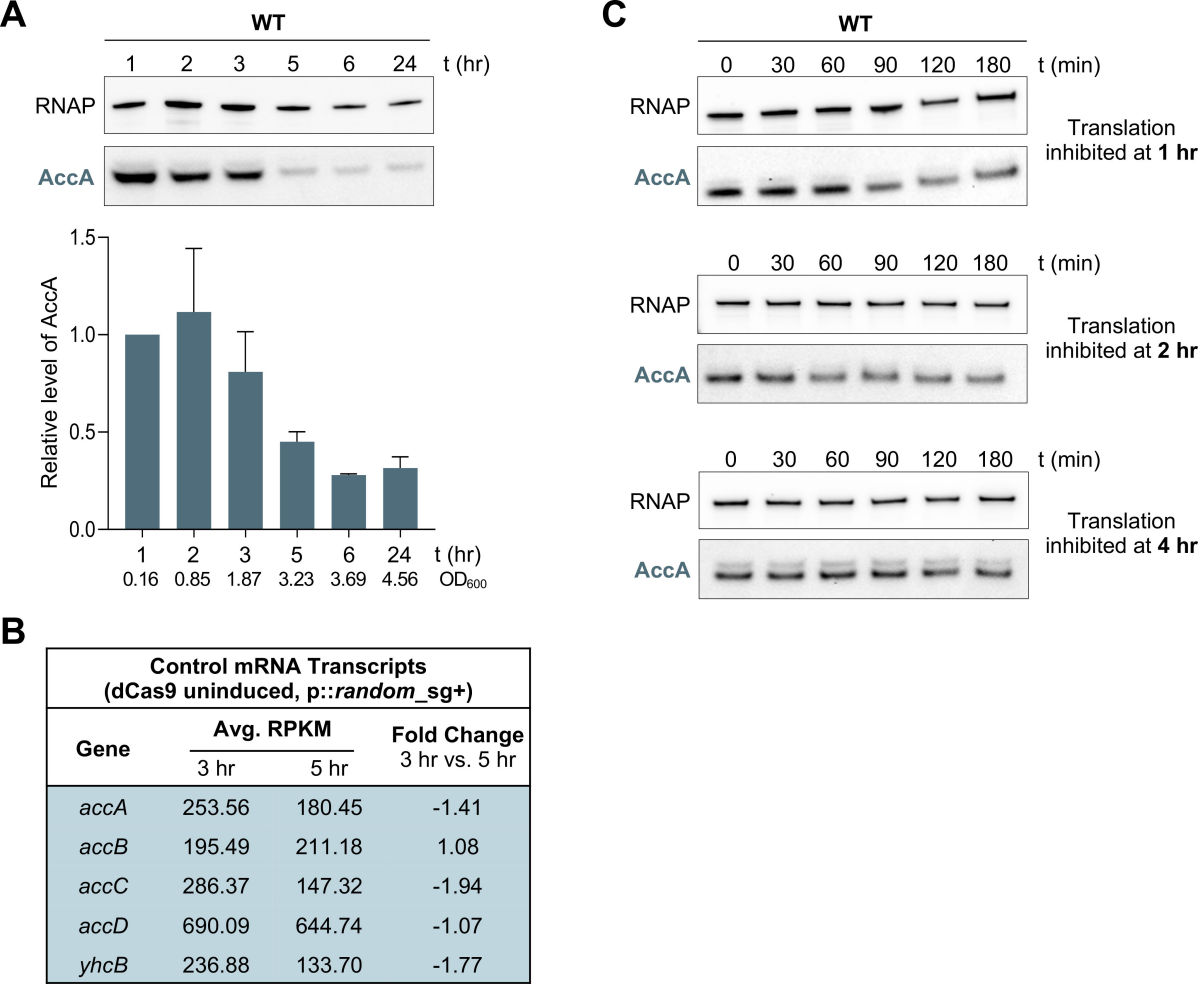
AccA levels are growth-phase regulated through proteolysis. (**A**) Steady-state assay of AccA abundance in wild type at 1, 2, 3, 5, 6, and 24 h post-inoculation. Samples were normalized by total protein (3 µg per lane) and analyzed by Western blot. AccA was detected with anti-His antibody, and RNAP (loading control) was detected using anti-RNAP antibody. AccA abundance was quantified by densitometry, normalized to RNAP, and expressed relative to AccA levels at the 1-h mark. Representative OD_600_ measurements of WT (AccA-His) are provided for each timepoint. (**B**) RNA-Seq of uninduced CRISPRi strains carrying the randomized small guide at 3 and 5 h. Reads per kilobase per million mapped reads and fold changes for *accA, accB, accC, accD,* and *yhcB* transcripts are shown. Data representative of biological triplicates. (**C**) Stability of AccA in the wild type was assessed at 1, 2, and 4 h post-inoculation. Representative starting OD_600_ measurements were 0.12 (1 h), 0.98 (2 h), and 2.50 (4 h). Translation was inhibited by the addition of 200 µg/mL chloramphenicol, and samples were collected at the indicated timepoints. Samples were normalized by total protein (3 µg per lane) and analyzed by Western blot. AccA was detected with an anti-His antibody, and RNAP served as a loading control. Data shown are representative of three biological replicates.

Our data indicate that the influence of YhcB on fatty acid flux is controlled by additional, post-transcriptional mechanisms (Fig. 2). Because YhcB interacts with AccA specifically during the transition into the stationary phase, we sought to determine whether this interaction affects the abundance or stability of AccA. A simple and attractive hypothesis we considered was that YhcB facilitates proteolytic turnover of AccA to downregulate ACC activity, perhaps functioning as an adapter for a protease. To investigate this possibility, we first examined the steady-state levels of chromosomally encoded AccA-His across the same time course used for the YhcB steady-state assays. Samples collected at the indicated timepoints were normalized by total protein and resolved by SDS-PAGE. AccA-His was visualized via Western blot, and the relative abundance of AccA was normalized to the loading control, RNAP (Fig. 5A).

In wild-type cells, AccA levels peak during exponential growth and decline markedly between 3 and 5 h, coinciding with the transition into stationary phase (Fig. 5A). Analysis of mRNA transcripts of WT (the uninduced, randomized CRISPRi strain) at 3 h compared to 5 h (Fig. 5B) revealed no significant change in *acc* gene transcripts, indicating the drop in AccA abundance results from post-transcriptional regulation. To distinguish between translational and proteolytic mechanisms, we measured AccA stability following inhibition of protein synthesis with chloramphenicol at 1, 2, and 4 h post-inoculation (Fig. 5C). Samples were collected at 0, 30, 60, 90, 120, and 180 min following translation arrest, normalized by protein content, and resolved by SDS-PAGE. AccA was visualized via Western blot, and RNAP was used as a visual loading control. During exponential growth (1 and 2 h), AccA abundance decreased progressively, demonstrating that the protein is actively degraded. However, AccA was stabilized when translation was inhibited at 4 h during the transition to the stationary phase. These findings indicate that AccA is subject to proteolytic turnover, but that its susceptibility to degradation changes across growth phases.

Although AccA is actively degraded during exponential growth, its high abundance at early timepoints indicates that synthesis outpaces degradation in this phase. As cells enter the transition to the stationary phase, AccA abundance decreases despite transcript stability and despite the later stabilization of the protein after 4 h. This pattern shows that proteolysis contributes to the decline in AccA abundance but cannot fully account for it, indicating that additional post-transcriptional mechanisms participate in controlling AccA levels. Collectively, these results establish that AccA abundance is governed by multiple growth-phase-dependent mechanisms, one of which is proteolysis.

### YhcB abundance affects AccA abundance

YhcB-AccA interactions occur during the transition to the stationary phase, a period when AccA abundance is reduced, and the protein is stabilized (Fig. 4A and B and 5C). This timing suggested that YhcB may influence AccA abundance. To test this possibility, we measured steady-state AccA levels in both Δ*yhcB* and the YhcB-overexpression strain across a similar time course used for monitoring AccA levels in wild type (Fig. 5A).

**Fig 6:**
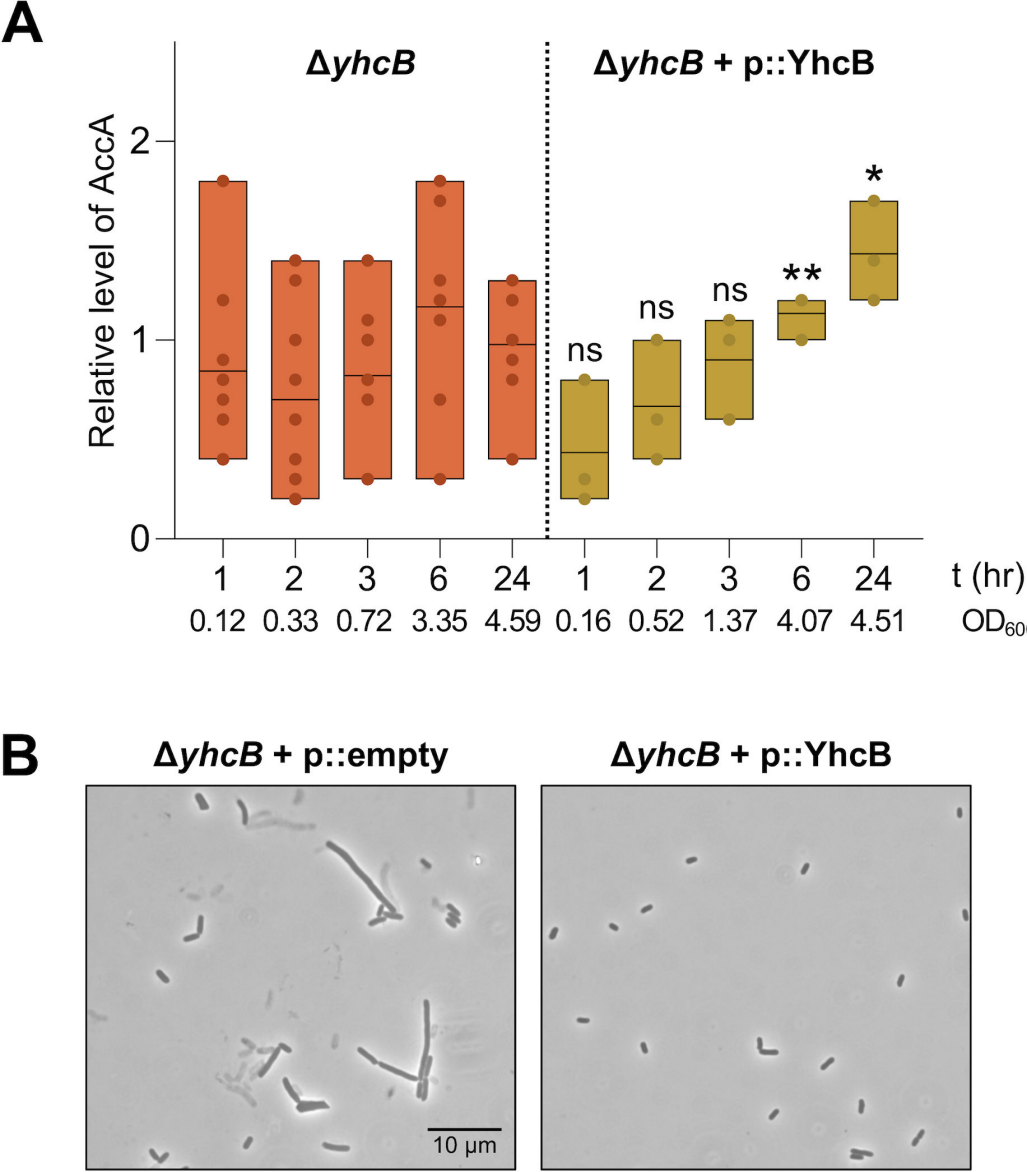
YhcB abundance affects AccA abundance. (**A**) Steady-state levels of AccA abundance in Δ*yhcB* and Δ*yhcB* + p::YhcB were assessed at 1, 2, 3, 6, and 24 h post-inoculation. Samples were normalized by total protein (3 µg per lane) and analyzed by Western blot. AccA was detected with anti-His antibody, and RNAP was detected as a loading control. AccA abundance was quantified by densitometry, normalized to RNAP, and expressed relative to AccA levels in wild-type cells at the 1-h mark. Representative OD_600_ measurements for each timepoint are provided. Significance between WT and the YhcB overexpression strain was assessed at each timepoint (e.g., WT 1 h vs. Δ*yhcB* + p::YhcB 1 h) using two-tailed, unpaired *t*-tests. NS indicates not significant; *P ≤ 0.05; and **P ≤ 0.01. Data shown represent nine biological replicates for Δ*yhcB* and three biological replicates for Δ*yhcB* + p::YhcB. (**B**) Phase-contrast microscopy at 1,000× magnification (10 µm scale bar) of the complemented *yhcB* mutant at 6 h. Overall, complementation using 0.05% arabinose resulted in normal cell morphology.

In the Δ*yhcB* background, AccA abundance was highly variable between biological replicates (*n* = 9) at all timepoints. Some replicates displayed markedly reduced AccA levels, whereas others resembled wild type. This variability suggests that the loss of YhcB leads to dysregulated AccA homeostasis rather than a uniform shift in AccA abundance (Fig. 6A). Attempts to measure AccA stability directly in the Δ*yhcB* were unsuccessful, as the strain is hypersensitive to both chloramphenicol and spectinomycin, making it impossible to reliably inhibit translation. Consequently, stability assays analogous to those performed in wild-type cells cannot be conducted in the Δ*yhcB* background, and our interpretation necessarily relies on steady-state abundance rather than protein half-life. Importantly, however, the lack of AccA stabilization in Δ*yhcB* indicates that YhcB is not required for AccA proteolysis. Instead, in the absence of YhcB, AccA turnover appears to proceed unchecked and may fluctuate depending on the metabolic state of the cell.

Conversely, overexpression of YhcB resulted in consistently elevated AccA levels in both early and late stationary phase (Fig. 6A). Despite increased AccA abundance, these cells displayed a cell morphology similar to wild type, indicating that ACC activity and FAB flux did not increase proportionally (Fig. 6B). This finding suggests that YhcB-bound AccA is stabilized but remains inactive or less accessible for incorporation into the ACC holoenzyme.

Taken together, these results demonstrate that YhcB influences AccA abundance in two distinct ways: loss of YhcB destabilizes AccA homeostasis, consistent with dysregulated turnover, whereas an overabundance of YhcB stabilizes AccA without increasing its activity. These outcomes are consistent with a model in which YhcB binding protects AccA from degradation yet simultaneously limits the ability of AccA to participate in ACC holoenzyme assembly, thereby modulating ACC activity during the transition to the stationary phase.

### ACC activity is regulated by multiple mechanisms

**Fig 7:**
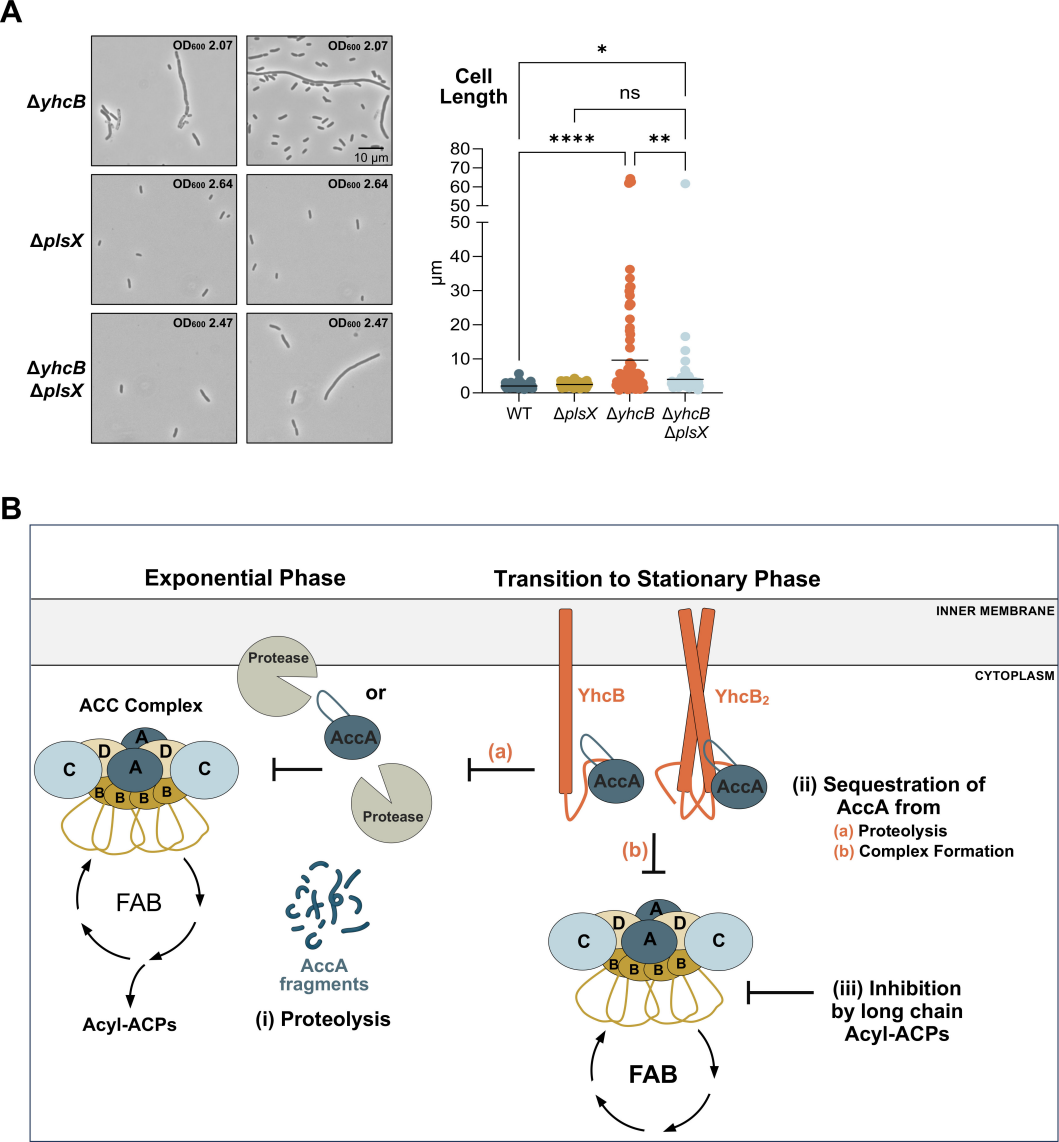
Model of proposed ACC regulation during the early stationary phase. (**A**) Phase-contrast microscopy at 1,000× magnification (10 µm scale bar) of *yhcB, plsX,* and *yhcB plsX* mutants at 5 h post back-dilution. Representative OD_600_ measurements at the time of microscopy for each strain are indicated in the top right of the micrographs. Two micrographs per strain are provided. Cell size analysis was performed on 92 cells per strain with MicrobeJ, and measurements were assessed using a one-way ANOVA test with Brown-Forsythe and Welch tests assuming that standard deviations were not equal. Significant differences were assessed using a Games-Howell test. NS indicates not significant; *P ≤ 0.05; **P ≤ 0.01; ***P ≤ 0.001; ****P ≤ 0.0001. Data shown are representative of a minimum of three biological replicates. (**B**) Three regulatory methods are proposed to act on the ACC complex (AccA_2_AccB_4_AccC_2_AccD_2_). (i) AccA is proteolytically degraded by an unknown protease whose activity is detectable in the exponential phase. (ii) YhcB levels peak in the early stationary phase, coinciding with YhcB-AccA interactions. These interactions may protect AccA from proteolysis and inhibit ACC complex formation through sequestration of AccA. (iii) As cells enter the stationary phase, stringent stress responses (e.g., ppGpp-mediated inhibition of PlsB) slow GPL synthesis, resulting in the accumulation of long-chain acyl-ACPs and feedback inhibition of ACC. Together, these regulatory mechanisms allow the cell to tightly control ACC activity commensurate with cellular resources and needs.

A well-established mechanism of ACC regulation is feedback inhibition by long-chain acyl-ACPs. To assess whether this method of regulation depends on YhcB, we utilized a genetic background in which feedback inhibition of FAB is known to occur. Cells lacking the phosphate acyltransferase PlsX accumulate long-chain acyl-ACPs, which inhibit FAB and reduce ACC activity (25). When we deleted *plsX* in Δ*yhcB*, a partial rescue of the characteristic filamentous morphology was observed (Fig. 7A). This partial suppression indicates that feedback inhibition of ACC is not dependent on YhcB and, importantly, that feedback inhibition of ACC alone cannot compensate for the loss of YhcB function.

The dysregulated AccA levels paired with the filamentous morphology seen in the *yhcB* mutant suggest that proteolysis and feedback inhibition together are insufficient to fully constrain FAB during the transition to the stationary phase. Although the molecular details of how YhcB modulates AccA remain unresolved, the data support a model in which YhcB provides a distinct, additional layer of ACC regulation to appropriately tune FAB flux during the crucial transition between growth phases.

## DISCUSSION

The ACC complex of *E. coli* has been a focus of biochemical and genetic research for over half a century. Foundational studies by multiple groups defined the architecture and function of the complex and firmly established its central role in supplying malonyl-CoA for type II FAB. Given ACC catalyzes the rate-limiting step of FAB, understanding how its activity is tuned to match growth phase and membrane demand has been an intense area of research, yet remains enigmatic. Several regulatory mechanisms have been proposed. Early work suggested a role for the stringent response alarmone ppGpp; however, subsequent studies showed that ppGpp does not directly regulate ACC. Instead, ppGpp binds to and inhibits PlsB, the glycerol-3-phosphate acyltransferase that commits acyl-ACPs to GPL synthesis (6, 26). Another proposed mechanism involves the transcriptional activator FadR because its overexpression activates cryptic, low-affinity promoter regions for the *acc* genes (in addition to most *fab* genes) and leads to overactive FAB. However, the levels of FadR required for this effect are not physiologically relevant under normal growth conditions (27–29). A third, well-supported mechanism is feedback inhibition, in which long-chain acyl-ACPs allosterically reduce malonyl-ACP production. This method of regulation is supported by both *in vitro* assays and LC-MS analyses that show long-chain acyl-ACP accumulation resulting in the reduction in malonyl-ACP levels (7, 30–32).

Still, close examination of work by Noga and co-authors (7) suggests that neither feedback inhibition of ACC alone nor slowed GPL flux via ppGpp-PlsB interactions can fully explain growth-phase regulation of ACC. In their study, when translation was inhibited in a *relA*-deficient strain (lacking the primary source of ppGpp), malonyl-ACP levels still decreased over time despite the absence of ppGpp. The only long-chain acyl-ACP that accumulated during the experimental time course was C14:0-ACP, which was proposed to feed back to inhibit ACC. However, the decrease in malonyl-ACP occurred more rapidly than C14:0-ACP accumulated, suggesting the involvement of additional regulatory inputs. Moreover, because SpoT-derived ppGpp production is likely a downstream consequence of FAB limitation rather than its cause, the ppGpp-dependent signaling pathway cannot fully account for the observed regulation (7, 33, 34). Together, these findings suggest additional regulatory mechanisms that modulate ACC activity in response to the growth phase.

While *E. coli* YhcB has been implicated in cell division, peptidoglycan synthesis, and even LPS regulation (14, 15, 35), our previous work established that loss of YhcB results in severe cell filamentation and stationary phase growth defects due to overactive FAB (12). Suppressor mutations in genes encoding the ACC complex or chemical inhibition of FAB fully reversed the *yhcB* phenotypes, directly linking YhcB to ACC function. Here, we clearly show that YhcB directly interacts with AccA, likely modulating ACC activity during the crucial exponential-to-stationary phase transition.

Analysis of LC-MS/MS peptide data from crosslinked His pulldowns of YhcB revealed several proteins related to FAB and FAD: AccA, AccC, AccD, FabD, FabH, FabI, and FadE (Fig. 3B). When previously investigating the *yhcB* mutant, we isolated several suppressors in the genes encoding subunits of the ACC complex. While this work focused on YhcB and AccA, whether YhcB interacts with additional lipid biosynthetic enzymes remains to be determined. Although other subunits of the ACC complex have not yet been studied in this context, Noga and co-authors (7) found that of the four subunits of the ACC complex, only AccA protein levels positively correlated with GPL flux. While these increases were small relative to GPL flux, they do demonstrate that AccA protein levels have a role in affecting downstream GPL synthesis and therefore affect FAB flux. Supporting this finding, an *accA* mutation (*accA*_R175L_) fully rescues the *yhcB* mutant, while a mutation in *accD* renders a partial rescue. Cell size was also reduced when the *accA*_R175L_ allele was transferred into WT, demonstrating that an AccA variant alone can slow FAB flux (12).

Assessment of YhcB steady-state levels throughout growth revealed that YhcB levels are highest during the transition out of the exponential phase, a pattern observed regardless of whether *yhcB* expression is controlled by its native promoter or an arabinose-inducible one (Fig. 4A and C). Not only are YhcB levels highest during the shift to stationary phase, YhcB-AccA interactions are also detected only during this transition (Fig. 4B). Conversely, AccA abundance normally peaks in exponential phase and drops sharply in early stationary phase (Fig. 5A). This reduction in AccA levels must be regulated post-translationally because *accA* transcripts do not significantly change across growth phases (Fig. 5B). Stability assays demonstrate that AccA is proteolytically degraded, detectable in exponential phase (Fig. 5C). AccA is stabilized when assessed in early stationary phase, coinciding with YhcB interactions, which suggests that YhcB interactions may protect AccA from proteolysis (Fig. 4B). Despite the increase in AccA abundance when YhcB is overexpressed, FAB flux is not overactive and supports the interpretation that YhcB-bound AccA is likely inactive (Fig. 6B).

We also report that the loss of YhcB causes dysregulation of AccA protein abundance, evidenced by highly varied steady-state levels across Δ*yhcB* replicates (Fig. 6A). The lack of transcriptional changes in *accA* expression in the CRISPRi *yhcB* knockdown strains or the *yhcB* mutant indicates that this dysregulation is post-transcriptional (Fig. 2C). Our data reveal that proteolysis is not dependent on YhcB, though it does respond to YhcB levels (Fig. 6A). When YhcB is absent, FAB flux is overactive and AccA abundance is variable across replicates. This variability may be the result of cell-to-cell adaptations due to dysregulated FAB flux caused by the loss of YhcB and may explain the pleomorphic cell morphology of the *yhcB* mutant (Fig. 1B, 6B, and 7A).

Together, these results support a model in which YhcB sequesters AccA during the transition from exponential to stationary phase, possibly reducing ACC complex assembly and slowing the initiation of FAB. This metabolic shift occurs at a time when FAB flux must be curtailed in response to reduced nutrients and activation of the stringent stress cascade—conditions that require reduced lipid synthesis and membrane biogenesis. The partial rescue of Δ*yhcB* by *plsX* deletion indicates that accumulation of long-chain acyl-ACP, which are the primary effectors of feedback inhibition, is not sufficient to fully suppress ACC activity (Fig. 5C). This observation points to an additional regulatory mechanism to modulate the initial steps of FAB during this growth phase transition.

In a wild-type cell, AccA protein levels decrease between 3 and 5 h before stabilizing sharply, and AccA is degraded in the exponential phase before stabilizing in the transition to the stationary phase (Fig. 5). YhcB-AccA complexes are detected only when YhcB is abundant, suggesting that complex formation may occur whenever both proteins are present at sufficient levels rather than requiring a dedicated activation signal (Fig. 4). This correlation between protein abundance and AccA interaction is consistent with growth-phase regulation of YhcB. It has been suggested that *yhcB* expression is negatively regulated by SdsR, a σ^s^-dependent small RNA (23, 24). SdsR has also been reported to regulate *tolC* and *mutS* transcription in *E. coli* and targets multiple genes in *Salmonella*, including *ompD, crp, stpA,* and *tolC* (23, 36–38). Its expression is limited to and is induced by σ^s^, a stress factor involved in stationary-phase regulatory cascades. This regulation mediated by SdsR in the stationary phase may explain the reduction in YhcB levels in that phase of growth, as seen in the 24-h timepoint (Fig. 4A) (36). However, the growth-phase control of YhcB in the exponential phase remains unclear.

We propose that three regulatory methods act on the ACC complex (Fig. 7B). In the exponential phase, AccA levels are high (Fig. 5A), and FAB flux is constant. In the first proposed method of regulation (Fig. 7B, i), AccA is degraded in this phase of growth. Protein levels of AccA drop significantly from 3 to 5 h, irrespective of transcript levels (Fig. 5), and this proteolytic turnover is mediated by an as-yet unidentified soluble or membrane-associated protease. YhcB levels are low but increasing throughout the exponential phase (Fig. 4 and 5). As the cell transitions to the stationary phase, our data suggest that YhcB binds AccA, potentially reducing the formation of active ACC holoenzymes and slowing the initiation of FAB (Fig. 7B, ii). The observation that AccA abundance, but not activity, increases with overexpressed YhcB suggests that YhcB acts to sequester AccA (Fig. 6). This sequestration may also protect AccA from the first proposed method of regulation, proteolysis (Fig. 7B, i). The final method of regulation (Fig. 7B, iii) is the canonical route of feedback inhibition, likely induced by ppGpp-PlsB interactions that slow GPL synthesis, in which long-chain acyl-ACPs inhibit ACC activity. Our work highlights two unique mechanisms the cell employs in a multifaceted approach to control ACC activity, ensuring lipid synthesis is tuned for optimal membrane biogenesis.

## MATERIALS AND METHODS

### Strains and growth conditions

All strains, plasmids, and primers used in this study are provided in Data Set S1. Unless otherwise stated, strains were grown in lysogeny broth (LB) or on LB agar at 37°C. Where indicated, growth media were supplemented with ampicillin (100 µg/mL), chloramphenicol (30 µg/mL or 200 µg/mL), hygromycin (100 µg/mL), kanamycin (30 µg/mL), anhydrous tetracycline (aTc) (200 nM), L-arabinose (0.05% [wt/vol]), IPTG (1 mM), and/or *p*-benzoyl-L-phenylalanine (*p*Bpa) (1 mM).

### Strain construction

TaKaRa ExTaq polymerase or Apex Taq RED polymerase was used for PCRs <5 kb for cloning or screening experiments, respectively. PFU Turbo polymerase AD (Agilent) was used for PCRs >5 kb. For all cloning, inserts and vectors were digested with the restriction enzymes (NEB) indicated in primer names. Ligations were performed with T4 DNA Ligase (NEB) and transformed by electroporation into DH5α-competent cells. To construct pMMB67EHhph, the backbone of pMMB67Ehkan (39) was amplified with primers XhoI-pMMBKn-for and SpeI-pMMBKn-rev. The *hph* resistance cassette was amplified from transposon T101 with the primers SpeI-hph-for and XhoI-hph-rev (40). After ligation and transformation, the resulting vector (pMMB67EHhph) was sequence confirmed. The insert for pINHIB2-swap, containing a *ptac* promoter, SwaI cut site for adding guide sequences, *cas9* handle, and Rho-independent terminator, was gene synthesized by GenScript. The synthesized insert was cut at flanking PacI cut sites, and the correctly sized product was gel extracted (Qiagen, QIAquick Gel Extraction Kit) from a 0.8% agarose gel. The pMMB67EHhph vector was amplified with pacI-pMMB-for and pacI-pMMB-rev and cut with PacI and DpnI. Vector and insert were ligated, transformed, and sequence confirmed. To add a *lacO* operator to pINHIB2-swap, the vector was amplified with BamHI-lacO-pINHIB2-for and BamHI-pINHIB2-rev. The whole-plasmid PCR product was cut with DpnI and BamHI and circularized by T4 DNA ligation. The resulting vector was named pINHIB3-swap (pINHIB).

Following design recommendations from Cui and co-authors (16), a small guide targeting the promoter region of *yhcB* was designed and cloned into pINHIB. The control guide of randomized nucleotides was cloned using Cui and co-authors’ control guide sequence of 5′-TGAGACCAGTCTAGGTCTCG-3 (16). The chromosomal strain of W3110 *dcas9* was generated using generalized phage transduction and the AV04 strain (Addgene #115926) (16). To link a selectable allele with *dcas*9, *yfjW* (close to the primary 186 *attB* site and with a predicted unrelated function) was deleted using generalized transduction, and phage lysate was made from the resulting strain. *dcas9* linked to Δ*yfjW* was then moved into W3110 via generalized phage transduction.

The chromosomal strains with tagged variants of YhcB and AccA were generated at native loci via the λ Red recombinase system in the DY330 strain as previously described (41, 42). The *p*Bpa variants of YhcB were first constructed in a vector through site-directed mutagenesis, which was used in plasmid-based His pulldowns. Site-directed mutants of a vector carrying *p*Bpa variants of YhcB-HA were used as a recombineering template for the generation of the chromosomal strain in DY330. Phage transduction was used to move selectable alleles from the donor DY330 strains to the recipient strains. Vectors were whole plasmid sequenced at Plasmidsaurus. Strains were verified via PCR confirmation, and specific strains were whole-genome sequenced (SeqCenter, LLC) (see Data Set S1).

Chromosomal deletions were generated in the *E. coli* K-12 strain W3110 via generalized transduction and the Keio collection (43). The flippase recognition target-flanked kanamycin cassette was removed by flippase site-specific recombination as previously described (44). When necessary, pCP20, which expresses the flippase from a temperature-sensitive promoter, was transformed into the strains possessing the kanamycin resistance cassette. Transformants were grown at 30°C overnight and then passaged at 37°C. Colonies were screened for loss of both kanamycin and ampicillin resistance, and the removal of the kanamycin cassette was confirmed via PCR amplification of the appropriate region (see Data Set S1) (44).

### CRISPRi knockdown of gene expression

Strains with a chromosomal copy of *dcas9* under the control of an aTc-inducible promoter and a vector carrying a small guide targeting *yhcB* or a randomized guide under the control of an IPTG-inducible promoter were back-diluted to an OD_600_ of 0.05 either with 1 mM IPTG (*yhcB* sg +) or without (*yhcB* sg −). Expression of *dcas9* was induced with 200 nM aTc at the times designated in the figure legends. Strains were then visualized via microscopy or harvested for RNA-Seq preparation.

### RNA-Seq and analysis

Strains with a chromosomal copy of *dcas9* under the control of an aTc-inducible promoter and a vector carrying either a randomized small guide or a small guide targeting *yhcB* under the control of an IPTG-inducible promoter were back-diluted to an OD_600_ of 0.05 with 1 mM IPTG (*yhcB/Rdm* sg +). Expression of *dcas9* was induced with 200 nM aTc at 3 and 5 h. One milliliter of each culture was collected at 10 min, and an additional 1 mL was collected after 1 h from the cultures where the small guide was induced at 5 h. One milliliter of culture was vortexed with 2 mL of RNAprotect (QIAGEN) for 15 s and incubated at room temperature for 5 min. Samples were then centrifuged at 5,000 × *g* for 10 min, and the supernatant was discarded. Samples were prepared in biological triplicate. Library preparation, rRNA depletion, and RNA sequencing were carried out by SeqCenter, LLC, using Illumina RNA Sequencing with ~12 M RNA reads/sample. RNA-seq analysis was performed using QIAGEN CLC Genomics Workbench.

### Affinity purification

Strains possessing the pEVOL::*p*Bpa tRNA synthetase (Addgene #: 31190) (19) and either the p::YhcB-*p*Bpa-His plasmids or a chromosomal copy of His-tagged AccA and HA-tagged YhcB-*p*Bpa variants under the control of the native promoter were back-diluted to an OD_600_ of 0.05. Cells were grown in 50 mL of LB with 0.05% arabinose and 1 mM *p*Bpa. At the specified times (2, 5, or 24 h), samples were irradiated in a 150 mm petri dish using the top of a UV light box (low setting, 302 nm, 25 W, UVP Transilluminator PLUS TFM-40V, Analytik Jena) at a volume depth of 6 mm for 10 min. Cell pellets from strains harboring p::YhcB-*p*Bpa-His plasmids were resuspended and rotated at room temperature for 20 min in 5 µL/mg BugBuster (Novagen), 200 µg/mL lysozyme, and 250 U/mL benzonase nuclease (Millipore Sigma). The whole-cell lysate was centrifuged at 16,000 × *g* for 20 min at 4°C to remove debris, and HEPES to 50mM, NaCl to 500 mM, glycerol to 10%, and cOmplete EDTA-free protease inhibitor (Roche) (1 tablet/10 mL lysate) were added to each lysate. Samples were solubilized at 4°C for 2.5 h and centrifuged prior to loading the supernatant onto a gravity column.

Cell pellets from strains harboring the His-tagged AccA were resuspended in 3.7 mL of lysis buffer A (50 mM HEPES [pH 7.5], 500mM NaCl, and 10% glycerol) and French pressed at 20,000 psi in the minicell pressure cell (GlenMills). The cOmplete EDTA-free protease inhibitor (Roche) (1 tablet/10 mL lysate) was added to each lysate immediately upon exiting the pressure cell, and the lysates were centrifuged at 5,000 × *g* for 20 min at 4°C. The cell-free supernatant was then centrifuged at 100,000 × *g* for 1 h at 4°C. The membrane pellet was solubilized in buffer B (buffer A containing 0.2% Triton X-100 and 20 mM imidazole) for 2.5 h and centrifuged.

Lysates from strains harboring p::YhcB-*p*Bpa-His plasmids or the chromosomal His-tagged AccA were loaded onto a 0.5 mL HisPur Ni-NTA column pre-equilibrated in buffer B, and the flow-through was collected. The column was washed with five column volumes of buffer B and collected. Finally, bound proteins were eluted using buffer C (buffer A containing 200 mM imidazole, 0.2% Triton). It was determined that 200 mM imidazole was optimal for elution of the target protein complexes. Eluted proteins were precipitated overnight at 4°C with TCA at 25% of the total volume. Samples were then subjected to protein fingerprinting via LC-MS/MS or visualized using immunoblotting. Because individual ACC proteins interact in a larger complex, this same experiment was also performed in the presence of 5 M urea with no change in the results.

### Mass spectrometry and proteomics analysis

Proteomic mass spectrometry analysis was performed by the University of Georgia Proteomics and Mass Spectrometry Core facility. Proteins were digested with trypsin and analyzed on the Orbitrap mass spectrometer (Thermo Scientific, Orbitrap Velo Elite) coupled with nano-HPLC using a 60-min elution gradient. Peptides were then searched against an *E. coli* protein database for identification. Proteins present in the crosslinked samples, the un-crosslinked control, and the empty vector control were excluded from analysis.

### Immunoblotting

Flow-through and wash samples (5.6 µL) were diluted with 4× LDS loading buffer, and the TCA-precipitated elution samples were resuspended in 10 µL of 1× LDS loading buffer. Purified crosslinked products were resolved by SDS-PAGE on a 10% Bis-Tris gel. Proteins were transferred to polyvinylidene difluoride transfer membrane (Amersham Hybond, Cytiva Life) at 15 V for 1 h. Proteins were visualized by Western blot using a conjugated His-tag antibody (6× His-Tag Antibody, Dylight 800, mouse, Rockland Immunochemicals) and an unconjugated anti-HA mouse antibody (THE HA Tag, Genscript) paired with anti-mouse Cyanine5 secondary antibody (Invitrogen). A purified anti-*Escherichia coli* RNA polymerase (RNAP) β mouse antibody (Biolegend) (paired with the anti-mouse Cyanine5 secondary) was used for the detection of RNAP as a loading control. Blots were visualized using the Biorad ChemiDoc MP Imaging System, and relative protein values were calculated using densitometry of the bands normalized to the RNAP loading control. A two-tailed, unpaired *t*-test was performed to determine significant differences between samples.

### AlphaFold 3 analysis

Protein interactions were modeled using AlphaFold 3 (20, 21). Model analysis was performed using UCSF ChimeraX and the structure prediction tool with the AlphaFold error plot (22). Interactions predicted within 5 Å were examined.

### Steady-state assays

Strains were normalized to an OD_600_ of 0.05 and grown for 24 h in 50 mL LB. Aliquots at each timepoint were taken at 1, 2, 3, 5, 6 (for YhcB levels and AccA levels in wild type only) and 24 h. Samples were pelleted at 5,000 × *g* at 4°C and immediately frozen at −20°C. Pellets were resuspended in 1× PBS-1% SDS lysis buffer and boiled for 10 min at 95°C. Samples were centrifuged at 17,000 × *g* for 20 min, and the supernatant was transferred to a new tube. Protein abundance was quantified using the Pierce Detergent Compatible Bradford Assay kit following the manufacturer’s instructions. Samples were normalized to load 1.5 µg of total protein for YhcB blots and 3 µg of total protein for AccA blots.

### Stability assays

Strains were normalized to an OD_600_ of 0.05 and grown for 1, 2, or 4 h in 50 mL LB. At the times designated in the figures, 200 µg/mL chloramphenicol was added to each culture to halt translation. Samples were collected at 0, 30, 60, 90, 120, and 180 min following the addition of chloramphenicol, pelleted at 5,000 × *g* at 4°C, and immediately frozen at −20°C. Pellets were resuspended in 1× PBS-1% SDS lysis buffer and boiled for 10 min at 95°C. Samples were centrifuged at 17,000 × *g* for 20 min, and the supernatant was transferred to a new tube. Protein abundance was quantified using the Pierce Detergent Compatible Bradford Assay kit following the manufacturer’s instructions. Samples were normalized to load 3 µg of total protein.

### Phase-contrast microscopy and cell measurements

Cultures were grown for 5 h and spotted on 3% agarose pads. Images were captured with the Olympus CX43 equipped with an Olympus Infinity 355 camera in phase-contrast. Measurements were performed on >5 fields of view with MicrobeJ and analyzed in GraphPad Prism 10. In the case of Δ*yhcB*-like filamentation, manual measurements were required for the extremely filamentous cells that were not detectable by MicrobeJ. These cells (*n* = 80) were measured manually using the segmented-line tool function in ImageJ. A one-way ANOVA test was performed with Brown-Forsythe and Welch tests assuming that standard deviations were not equal, and significant differences were assessed using a Games-Howell test.

## Data Availability

Additional replicate data can be found in the Zenodo repository at doi 10.5281/zenodo.17792670.

## SUPPLEMENTAL MATERIAL

Data Set S1 mbio.03681-25-s0001.xlsx Description of strains, plasmids, primers, and RNA seq data.

Supplemental Material mbio.03681-25-s0002.pdf Figures S1-S5 and caption to Data Set S1.

ASM does not own the copyrights to Supplemental Material that may be linked to, or accessed through, an article. The authors have granted ASM a non-exclusive, world-wide license to publish the Supplemental Material files. Please contact the corresponding author directly for reuse.
