# Supplementary material for: YhcB mediates growth-phase control of fatty acid biosynthesis through regulation of acetyl-CoA carboxylase
Source: mBio. 2026 Jan 26;17(3):e03681-25. doi: 10.1128/mbio.03681-25 (PMC12977641; doi:10.1128/mbio.03681-25)
Supplement: Supplemental Material — Figures S1-S5 and caption to Data Set S1. [file mbio.03681-25-s0002.pdf]

Supporting materials supporting "YhcB mediates growth-phase control of fatty acid biosynthesis through regulation of acetyl-CoA carboxylase" by HM Stanley and MS Trent.

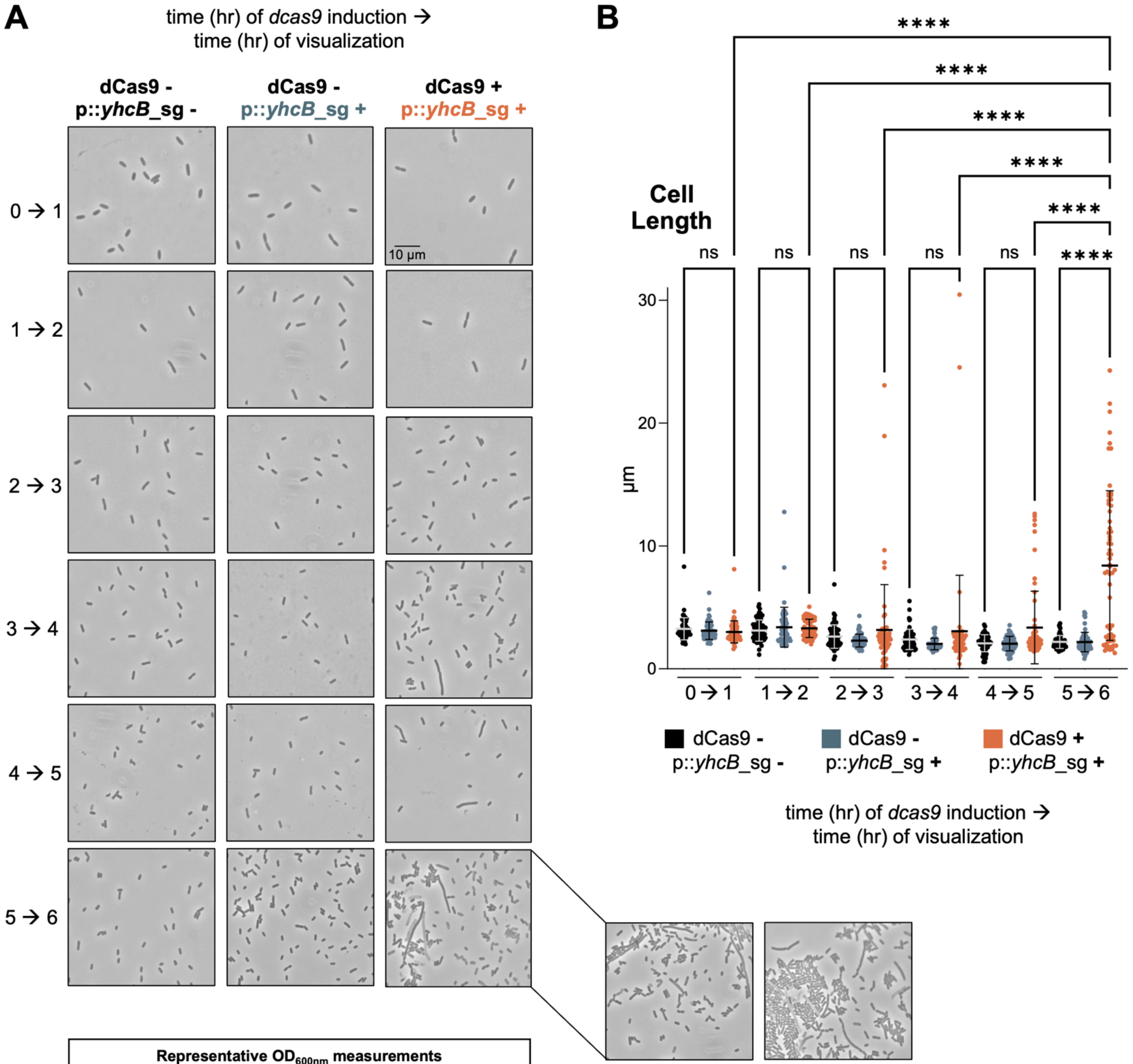

Representative OD_{600nm} measurements during time course

OD_{600} at time of microscopy	dCas9 - p::*yhcB*_sg -	dCas9 - p::*yhcB*_sg +	dCas9 + p::*yhcB*_sg +
1 hr	0.11	0.11	0.12
2 hrs	0.49	0.50	0.39
3 hrs	1.22	1.16	1.14
4 hrs	2.07	2.15	1.97
5 hrs	2.44	2.44	1.99
6 hrs	3.08	3.14	2.33

FIG S1 Additional microscopy analysis of controls and expanded time points of CRISPRi strains. A. Microscopy of cells during knockdown of *yhcB* expression across different phases of growth. Two additional

fields of view are shown for the final timepoint of the induced strain. Phase-contrast microscopy at 1,000x magnification (10-μm scale bar) is shown. Strains harbor a chromosomal copy of *dcas9* with an aTc-inducible promoter and a vector encoding a small guide targeting *yhcB* with an IPTG-inducible promoter. Cultures were back-diluted to OD_{600} 0.05 either with 1 mM IPTG (***yhcB* sg +**) or without (***yhcB* sg -**). Expression of *dcas9* was induced with 200 nM aTc at the times indicated. The numbers on the left side of each arrow indicate the hour when *dcas9* induction occurred (**dCas9 +/-**), and the numbers on the right of the arrow indicate the hour at which cells were imaged. OD_{600} measurements of the strains at time of microscopy are indicated in the table. Additional micrographs have been included for the **dCas9$^{+,}$ *yhcB* sg^{+}** strain (see 5→6 time point). **B.** Cell size analysis was performed on 63 cells per strain with MicrobeJ, and measurements were assessed through a one-way analysis of variance (ANOVA) test with Brown-Forsythe and Welch tests assuming that standard deviations were not equal. Significant differences were assessed using a Games-Howell test. NS indicates not significant; *, $P \leq 0.05$; **, $P \leq 0.01$; ***, $P \leq 0.001$; ****, $P \leq 0.0001$. Data shown are representative of 2 biological replicates.

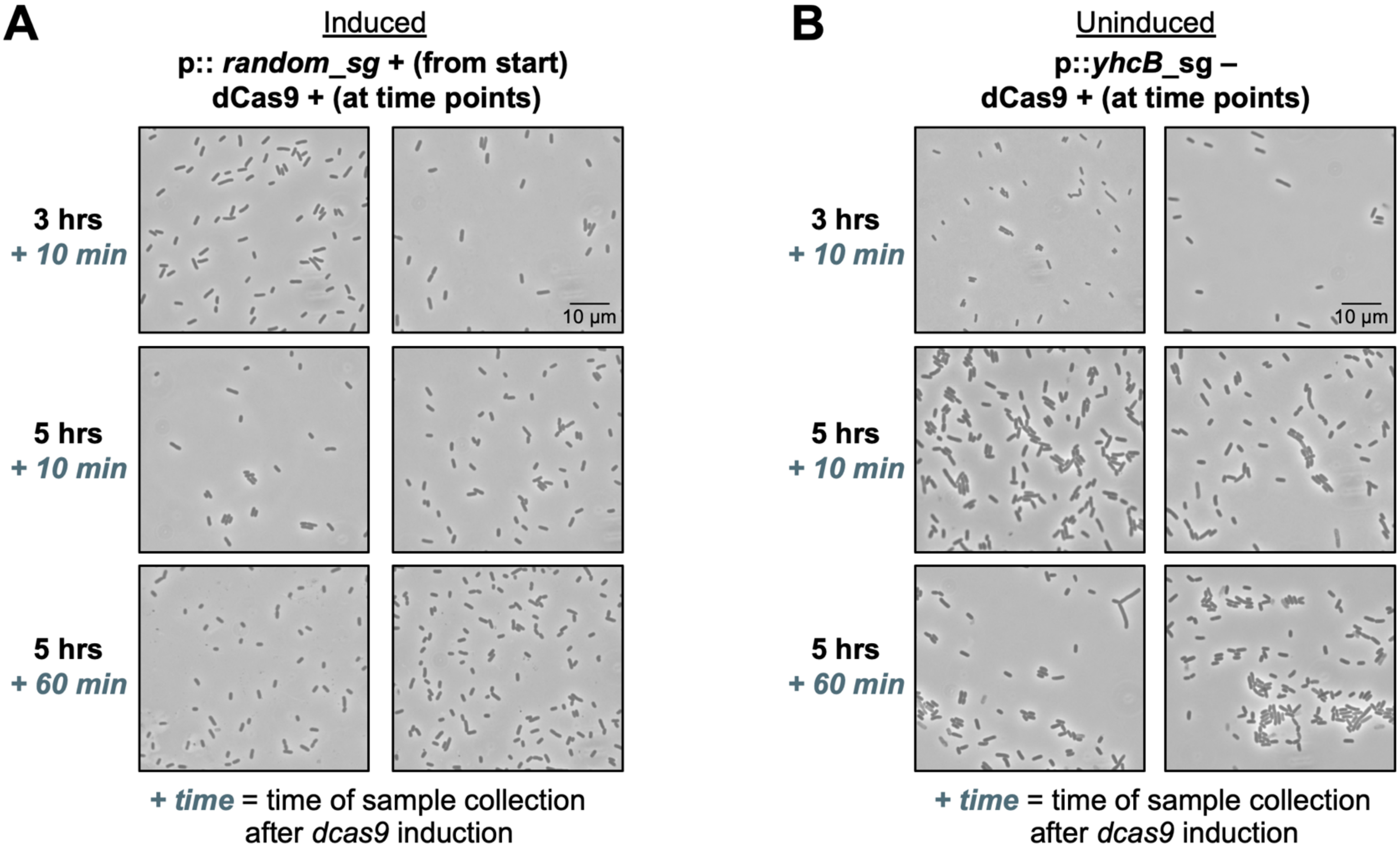

**Representative OD_{600nm} measurements during time course**	
OD_{600} at time of microscopy	p:: *random_sg* + (from start) dCas9 + (at time points)
**3 hrs** ***+ 10 min***	1.26
**5 hrs** ***+ 10 min***	2.56
**5 hrs** ***+ 60 min***	2.93

**Representative OD_{600nm} measurements during time course**	
OD_{600} at time of microscopy	p::*yhcB*_sg – dCas9 + (at time points)
**3 hrs** ***+ 10 min***	1.36
**5 hrs** ***+ 10 min***	2.63
**5 hrs** ***+ 60 min***	3.08

FIG S2 Additional microscopy analysis of controls included in CRISPRi RNASeq. Phase-contrast microscopy at 1,000x magnification (10-µm scale bar). Each strain contains a chromosomal copy of *dcas9* with an aTc-inducible promoter and a vector carrying a small guide targeting *yhcB* or a randomized small guide with an IPTG-inducible promoter. Cultures were back-diluted to OD_{600} 0.05 either with 1 mM IPTG (***yhcB* sg +**) or without (***yhcB* sg -**). Expression of *dcas9* was induced with 200 nM aTc at the times indicated. Numbers on the left side of the micrographs indicate the hour when *dcas9* induction occurred, and the italicized numbers indicate the hour when cells were imaged and harvested for RNASeq. Panel **A** shows micrographs of the induced randomized small guide control. Panel **B** depicts micrographs of the uninduced *yhcB*-targeting small guide strain. Representative OD_{600} measurements are shown in the tables. Data shown are representative of a minimum of 3 biological replicates.

A

Identify large or aromatic residues in YhcB to replace with *p*Bpa (n=14)

↓

Assess plasmid-based complementation of *p*Bpa variants of YhcB in Δ*yhcB* mutant

↓

Complementation Phenotype of YhcB-*p*Bpa Variants

Toxic	No Rescue	Partial-to-Full Rescue	
*p*Bpa6:F56	*p*Bpa2:Y5	*p*Bpa1:W3	***p*Bpa9:Q75**
	*p*Bpa3:F23	***p*Bpa4:Y36**	*p*Bpa10:H76
		*p*Bpa5:H55	*p*Bpa11:E87
		*p*Bpa7:H68	***p*Bpa12:F95**
		*p*Bpa8:Y74	***p*Bpa13:Q108**
		*p*Bpa14:Y117	

B

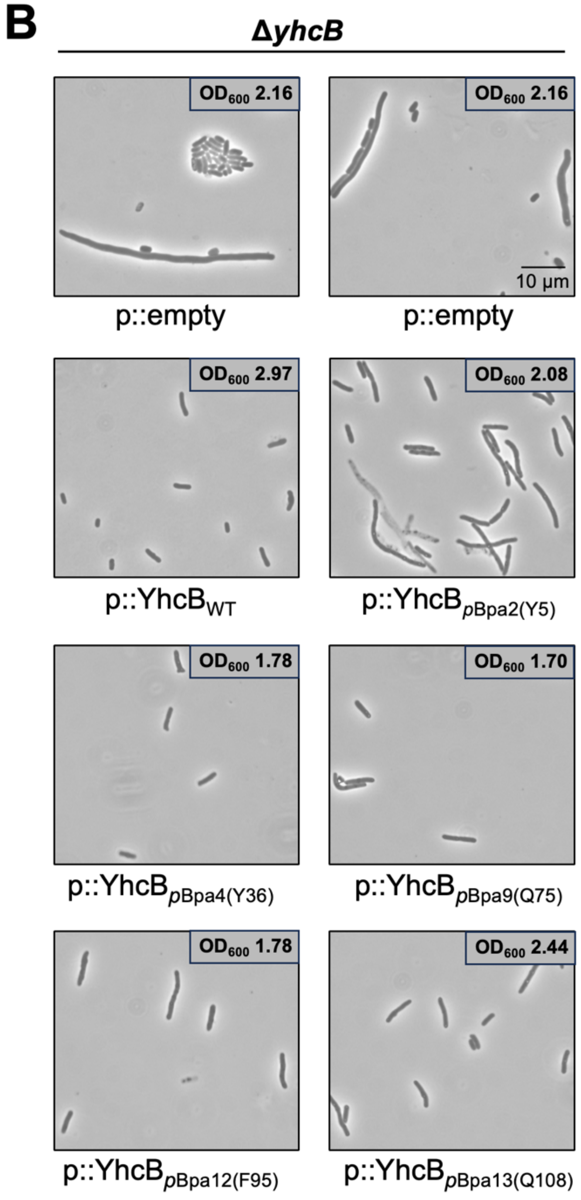

C

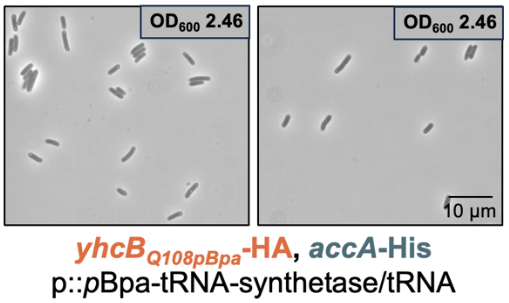

FIG S3 Generation of YhcB-*p*Bpa variants and assessment of their function by microscopy. A. Large or aromatic residues were identified in YhcB (n=14) and replaced with the artificial amino acid *p*Bpa. Plasmid-based complementation in the *yhcB* mutant was assessed via microscopy, and variants were ranked based on their ability to restore normal cell morphology. Four variants were chosen for downstream experiments. **B.** Phase-contrast microscopy (1,000x magnification; 10-μm scale bar) of Δ*yhcB* strains complemented with plasmid-encoded YhcB variants at 5 hrs. Representative OD_{600} measurements are indicated in the top right of the micrographs and two micrographs are shown for Δ*yhcB* + p::empty. **C.** Phase-contrast microscopy (1000x magnification; 10-μm scale bar) of the chromosomally tagged *yhcB*$_{Q108pBpa}$-HA and *accA*-His strain (used in **Fig. 3D** and **4B**) at 5 hrs. Data shown are representative of 2 biological replicates.

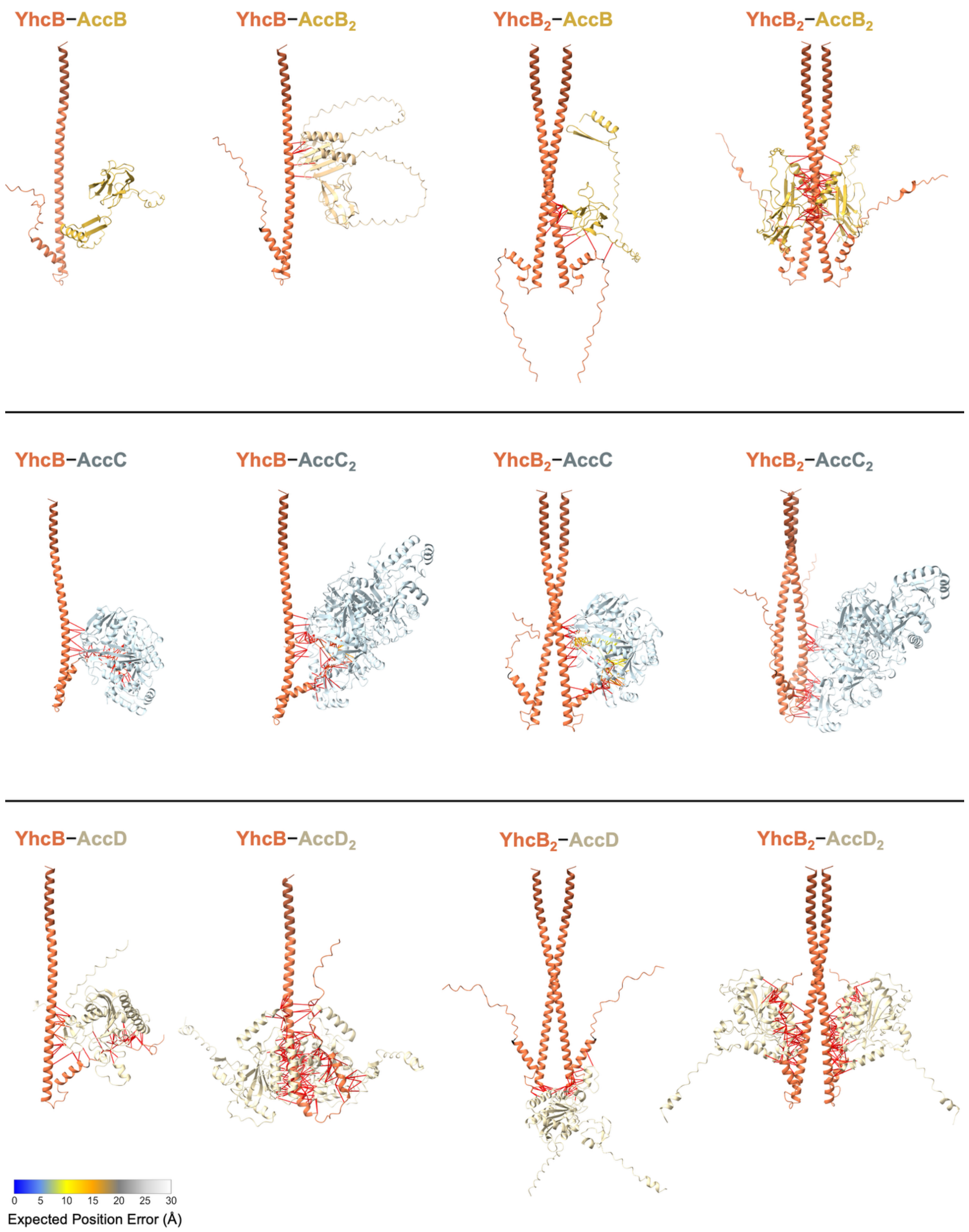

FIG S4 AlphaFold 3 models of YhcB and AccB, AccC, and AccD and predicted interactions at 5 Å or less. Scale bar indicates expected position error in Å of the predicted interactions where blue suggests strong confidence and white indicates weak confidence.

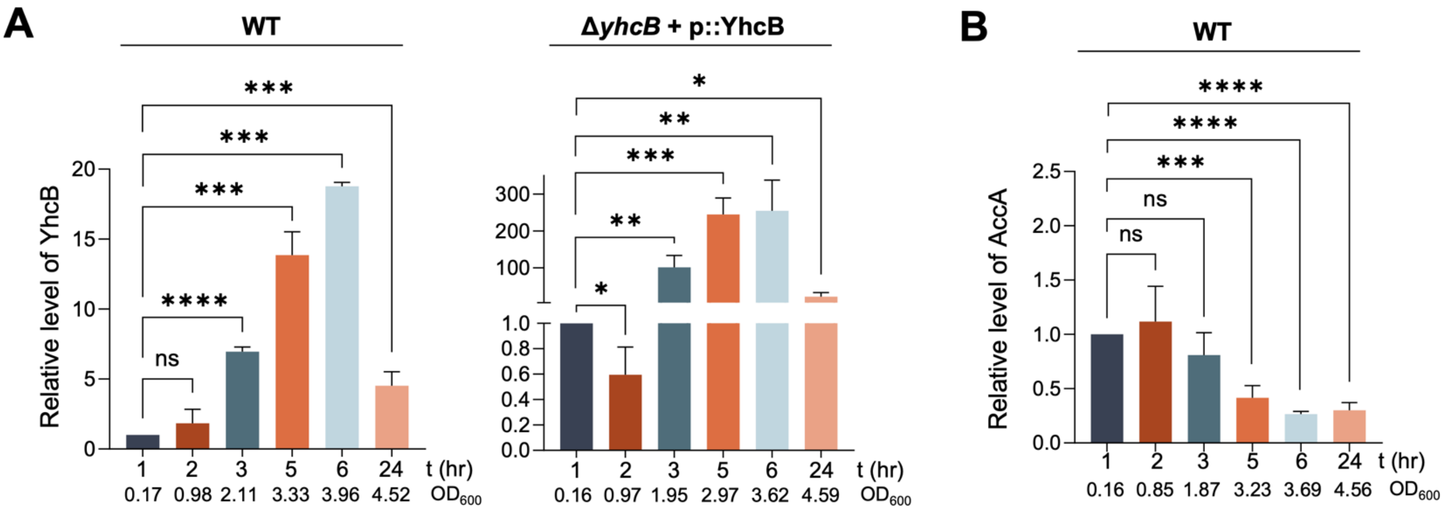

FIG S5 Additional statistical assessment of steady-state assays. A. Steady-state assay of YhcB abundance in WT and in Δ*yhcB* expressing *yhcB* from a vector under the control of an arabinose-inducible promoter (0.05% arabinose). YhcB abundance was assessed at 1, 2, 3, 5, 6, and 24 hrs post-inoculation. Samples were normalized by total protein (1.5 µg per lane) and analyzed by Western blot. YhcB was detected using anti-His antibody, and RNAP was detected with an anti-RNAP antibody. YhcB abundance was determined as described in **Fig 4** with hr 1 set as relative 1.0 for each graph. **B.** Steady-state assay of AccA abundance in WT was assessed at 1, 2, 3, 5, 6, and 24 hrs post-inoculation. Samples were normalized by total protein (3 µg per lane) and analyzed by Western blot. AccA was detected with anti-His antibody, and RNAP was detected with an anti-RNAP antibody. AccA abundance was determined as described in **Fig 5**. Representative OD_{600} measurements for time points in both figure panels are indicated. Significance for both panels was assessed using two-tailed, unpaired T-tests. NS indicates not significant; *, $P \leq 0.05$; **, $P \leq 0.01$; ***, $P \leq 0.001$; ****, $P \leq 0.0001$. Data shown are representative of a minimum of 3 biological replicates.

Data Set S1 (separate file). Listing of key reagents (strains, plasmids, and primers) and RNASeq data. Tab 1 provides a list of strains and plasmids used in this study. Strains subjected to whole-genome sequencing have been indicated. Primers used in this study are listed in Tab 2. Tab 3 shows the RNASeq data set of CRISPRi knockdown of *yhcB* expression (compared to randomized small guide control) and the *yhcB* mutant (compared to wild type). Tab 4 provides the RNASeq data set of uninduced randomized small guide control RPKMs at time points 3 hrs + 10 min, 5 hrs + 10 min, and 5 hr + 60 min.

DATA AVAILABILITY

Additional replicate data can be found in the Zenodo repository (**10.5281/zenodo.17792670**)
